# Concomitant Sjögren's Syndrome Was Not Associated with a Poorer Response or Outcomes in Ursodeoxycholic Acid-Treated Patients with Primary Biliary Cholangitis

**DOI:** 10.1155/2019/7396870

**Published:** 2019-06-02

**Authors:** Ping Ni, Ruoting Men, Mengyi Shen, Tingting Wang, Chen Huang, Xiaoli Fan, Li Yang

**Affiliations:** Department of Gastroenterology & Hepatology, West China Hospital, Sichuan University, Chengdu, Sichuan 610041, China

## Abstract

**Aim:**

Patients with primary biliary cholangitis (PBC) have at least 60% probability of having an autoimmune extrahepatic condition, with the most common being Sjögren's syndrome (SS). The impacts of SS on the response and outcomes in ursodeoxycholic acid (UDCA)-treated patients with PBC, however, remain unclear. The aim of this study was to document the biochemical responses and clinical outcomes of UDCA-treated patients with concomitant SS and to compare the findings to those of patients with PBC alone.

**Methods:**

Data from consecutive patients with PBC who visited West China Hospital affiliated with Sichuan University between October 2013 and October 2017 were reviewed retrospectively.

**Results:**

The study populations consisted of 226 patients with PBC alone and 56 with PBC/SS. The median ages, proportions of female patients, Fib-4 scores, and aspartate aminotransferase (AST)/platelet ratio index (APRI) at baseline in the two cohorts were similar. At presentation, patients with PBC/SS had higher serum IgG levels and positive rates for serum antinuclear antibody (ANA) than patients with PBC alone (all* P *< 0.05). There was no statistically significant difference between the rate of biochemical response to UDCA at 1 year in the PBC/SS and PBC alone groups. The UK-PBC risk scores and GLOBE scores in UDCA-treated patients in the two cohorts were also similar. During the follow-up period, the differences in the liver enzyme levels, Fib-4 scores, APRI, and incidence of liver-related adverse events were not significant.

**Conclusions:**

The results of this retrospective, single-center study suggest that the response and clinical outcomes of UDCA-treated patients with PBC are not adversely affected by concomitant SS.

## 1. Introduction

Primary biliary cholangitis (formerly called primary biliary cirrhosis, PBC) [1] is a liver disease characterized by destruction of the intralobular bile ducts that may eventually lead to cirrhosis and liver failure [2]. The pathogenesis of PBC remains largely unknown. PBC is assumed to result from a complex multistep process, in which different combinations of genetic and environmental factors interact [3]. PBC is mainly diagnosed in women, with female-to-male ratios of approximately 10 to 1 in the United Kingdom, North America, and Sweden [4] and 6.1 to 1 in China [5]. PBC is most often diagnosed when routine laboratory studies reveal an increase in alkaline phosphatase (ALP) levels. Antimitochondrial antibodies (AMAs), the serological hallmark of PBC, are present in most patients with the disorder. Histopathologically, PBC is characterized by portal inflammation and immune-mediated destruction of the intrahepatic bile ducts. Fatigue and pruritus are the most common presenting symptoms [6]. Ursodeoxycholic acid (UDCA), at a dose of 13 to 15 mg per kilogram of body weight per day, is the first-line drug for PBC. UDCA decreases serum liver enzyme levels and significantly reduces the likelihood of liver transplantation or death after four years [7, 8].

Sjögren's syndrome (SS) is a chronic inflammatory autoimmune disease with unknown etiology that often involves the lacrimal and salivary glands. Later in the course of the disease, other organs, such as the lungs, kidneys, liver, cardiovascular system, and central nervous system, are also involved. Dry eyes and dry mouth are the most common manifestations of SS [9]. SS can occur alone (primary SS, pSS) or in association with other autoimmune diseases (secondary SS, sSS). Patients with pSS were recruited for our research, and those with sSS were excluded.

Patients with PBC can have a variety of autoimmune diseases, such as SS, systemic lupus erythematosus (SLE), and rheumatoid arthritis (RA), with the most common being SS [10]. This study analyzed the clinical characteristics of PBC patients admitted to our hospital during the past 4 years and found that 19.9% of patients had PBC/SS. The impact of SS on the response and outcomes of UDCA-treated patients with PBC, however, remains unclear. Floreani et al. [11] found that associated SS did not affect the survival of PBC patients; however, a recent study [12] found that the overall survival rate was significantly higher in the PBC alone group than in the PBC/SS group. The aim of this study was to document the response and clinical outcomes of UDCA-treated patients with concomitant SS and compare the findings to those of patients with PBC alone.

## 2. Patients and Methods

### 2.1. Patient Population

This was a retrospective, single-center, data analysis of consecutive patients with PBC in the autoimmune liver disease (AILD) database we established in October 2013. All patients recruited had visited West China Hospital affiliated with Sichuan University between October 2013 and October 2017. The diagnosis of PBC [7] can be established when two of the following three criteria are met: (1) biochemical evidence of cholestasis based mainly on elevated ALP levels; (2) presence of AMA; and (3) histologic evidence of nonsuppurative destructive cholangitis and destruction of interlobular ducts.

PBC patients were considered to have concomitant SS if they had a total score of ≥4 for the following items [13]: anti-SS-related antigen A (SSA)/Ro antibody positivity and focal lymphocytic sialadenitis with a focus score of ≥1 foci/4 mm^2^, each scoring 3; an abnormal Ocular Staining Score of ≥5 (or van Bijsterveld score of ≥4), a Schirmer test result of ≤5 mm/5 min, and an unstimulated salivary flow rate of ≤0.1 mL/min, each scoring 1. Patients with overlapping syndromes (PBC plus autoimmune hepatitis or primary sclerosing cholangitis), chronic hepatitis B and C infections, drug-induced liver disease, hereditary hemachromatosis, Wilson's disease, IgG4-RD disease, alcoholic liver disease, and other causes of chronic liver disease were excluded.

All patients (PBC alone and PBC/SS) were treated with UDCA at a dose of 13-15 mg/kg/d, and 34% (19/56) of PBC/SS patients were also treated with glucocorticoids or immunosuppressant.

### 2.2. Laboratory Analyses

Given the decrease in exocrine secretions observed in SS, patients most commonly present with dry eyes and dry mouth. Liver enzyme testing included serum alanine aminotransferase (ALT) and aspartate aminotransferase (AST), ALP, and gamma-glutamyl transferase (GGT) levels. Liver function tests included serum total bilirubin and albumin levels and the international normalized ratio (INR) of the prothrombin time. The platelet count and serum creatinine and IgG and IgM levels were also recorded at the initial and/or follow-up visits. Rheumatoid factor, ANA, and AMA were also evaluated.

### 2.3. Biochemical Response

The biochemical response of patients after UDCA treatment for 12 months was assessed by the Paris-II definition (ALP and AST levels < 1.5× the upper limit of normal, and normal bilirubin level) [14], the Paris-I definition (ALP levels < 3× the upper limit of normal, AST levels < 2× the upper limit of normal, and normal bilirubin level) [15], the Toronto definition (ALP levels≤1.67× the upper limit of normal) [16], and the Barcelona definition (decrease in ALP levels > 40% or normal ALP level) [17].

### 2.4. Disease Progression and Outcomes

PBC disease progression was assessed by pretreatment serum ALP and GGT levels, Fib-4 scores, and percent of individuals with an AST/platelet ratio index (APRI). The UK-PBC score was determined to quantify the risk of future events for individualized risk prediction. Transplant-free survival could still be accurately calculated by the GLOBE score.

### 2.5. Other Extrahepatic Autoimmune (EHA) Diseases

In addition to SS, we also compared PBC alone with PBC combined with other extrahepatic autoimmune (EHA) diseases, such as rheumatoid arthritis (RA), systemic sclerosis (SSc), dermatomyositis (DM), and undifferentiated connective tissue disease (UCTD). Patients with other EHA diseases were also treated with UDCA at a dose of 13-15 mg/kg/d. The above methods were used to compare the differences between the PBC alone group and the PBC/other EHA diseases group.

### 2.6. Statistics

The descriptive statistics are presented as the median (interquartile range). Categorical variables are reported as counts and percentages. The differences in continuous variables among groups were compared with nonparametric tests. Categorical variables were compared using *χ*2-tests. All of the analyses in the current study were 2-tailed, and a* P*-value of <0.05 was considered significant. All analyses were computed using SPSS (SPSS version 23.0 for Windows, IBM Corp., Armonk, NY, USA).

The study was approved by the Ethics Committee of West China Hospital (No. 2013221). Methods were performed in accordance with the approved guidelines. All subjects provided written informed consent before enrolment.

## 3. Results

### 3.1. Patient Population

The study population consisted of 282 individuals. Of the 282 patients, 226 (80%) had PBC alone, and 56 (20%) had PBC/SS. As shown in Table 1, the median (interquartile range) age of patients with PBC alone was 52.5 (43.0, 62.0) years, while that of patients with PBC/SS was 51.5 (45.0, 58.8) years (*P *= 0.778). There was a greater proportion of females in the PBC/SS group (92.9%) than in the PBC alone group (83.6%); however, the difference was not significant (*P*=0.123). Seventy-one and 19 patients had splenomegaly in the PBC alone and PBC/SS groups, respectively, though the difference was not significant (*P* = 0.718).

### 3.2. Laboratory Analyses

As shown in Table 1, baseline serum IgG levels were significantly higher in the PBC/SS group than in the PBC alone group 19.3 (15.5, 22.2) g/L vs. 16.3 (14.2, 19.4) g/L (*P *= 0.001). Additionally, the proportion of patients who were positive for serum ANA was significantly higher in the PBC/SS group than in the PBC alone group (ANA: 56 (100.0%) vs. 210 (92.9%) (*P *= 0.041)). There were no differences in other laboratory analyses.

### 3.3. Biochemical Response

As shown in Table 2, according to the standard of care, the rates of the biochemical responses in the PBC and PBC/SS groups were not statistically significant (all* P* > 0.05) at month 12, regardless of the Paris I, Paris II, Toronto, or Barcelona definitions. In this study, 19 patients in the PBC/SS group were treated with glucocorticoids or immunosuppressants in addition to UDCA. The symptoms of dry mouth and dry eyes were severe in these 19 patients, and 7 of them were associated with joint pain. Excluding the 19 patients who were treated with glucocorticoids or immunosuppressants, 22 (59.5%), 25 (67.6%), 28 (75.7%), and 30 (81.1%) of the remaining 37 patients with PBC/SS who received UDCA alone for 1 year achieved a biochemical response according to the Paris I, Paris II, Toronto, or Barcelona definitions, respectively. The differences between the rate of biochemical response in the PBC and PBC/SS groups were still not statistically significant (*P* > 0.05).

### 3.4. Disease Progression and Outcomes

The median follow-up for patients with PBC alone was 21.0 (14.0, 38.3) months and 28.0 (11.0, 65.0) months for those with PBC/SS (*P *= 0.256). Serum ALP and GGT levels declined with the initiation of UDCA therapy in both groups. There were no differences in liver enzyme levels or function tests in either group at the end of the follow-up period. As shown in Table 3, the Fib-4 scores and APRI at the end of the follow-up period were higher in patients with PBC/SS than in those with PBC alone (Fib-4: 4.0 (1.9, 8.8) vs. 3.0 (1.8, 6.8), APRI: 1.0 (0.5, 2.1) vs. 0.9 (0.4, 1.6)). The difference in the Fib-4 scores and APRI did not reach statistical significance (all* P *>0.05). The UK-PBC score was determined to quantify the risk of future events for individualized risk prediction. Transplant-free survival could still be accurately calculated by the GLOBE score. Similarly, no significant association was found between the UK-PBC risk scores and GLOBE scores of the two groups (all* P* >0.05; Tables 5 and 6). Sixteen patients had liver-related adverse events (hepatocellular carcinoma, liver transplantation, and death) including 13 (5.8%) patients with PBC alone and 3 (5.4%) with PBC/SS (*P *=1.000; Table 4).

### 3.5. Other Extrahepatic Autoimmune (EHA) Diseases

A total of 7 patients with extrahepatic autoimmune diseases other than SS were included in this study. Of the 7 patients, 2 had PBC/RA, 2 had PBC/SSc, 1 had PBC/DM, and 2 had PBC/UCTD. As shown in Supplementary Tables 1 and 3, there were no differences in the data at baseline and the end of observation of the two groups except for serum albumin levels. As shown in Supplementary Table 2, the rates of the biochemical responses in the PBC and PBC/other EHA diseases groups were not statistically significant (all* P* > 0.05) at month 12.

As shown in Supplementary Tables 1 and 3, serum albumin levels were significantly higher in the PBC/other EHA diseases group than in the PBC alone group 45.9(40.5,47.7) g/L vs. 41.4(35.1,44.9) g/L (*P *= 0.039), 46.9(45.7,48.6) g/L vs. 42.8(35.0,46.5) g/L (*P *= 0.010) at baseline and at the end of observation. Compared with the PBC alone group, the PBC/other EHA diseases group had higher GLOBE scores and lower UK-PBC scores (*P* < 0.05) (Supplementary Tables 4 and 5). Additionally, no liver-related adverse events occurred in the PBC/other EHA diseases group during the follow-up.

## 4. Discussion

Contrary to the anticipated findings, the results of this study suggest that concomitant SS was not associated with a poorer response and outcomes in UDCA-treated patients with PBC.

Similar to PBC, SS is a common disease with a female-to-male ratio of at least 9:1 and a mean age at diagnosis of approximately 50 years [18]. Thus, the median age and proportion of females at baseline in the two cohorts were similar.

Hyperimmunoglobulinemia often occurs in SS, which is why serum IgG levels were significantly higher in the PBC/SS group than in the PBC alone cohort at baseline [18].

The Paris-II criteria have been designed specifically to better fit early-stage patients, who represent more than two-thirds of patients in recent cohorts [14]. We also used the Paris I, Toronto, and Barcelona definitions to evaluate biochemical responses. In this study, there was no statistically significant difference between the rate of biochemical response to UDCA at 1 year in the PBC/SS and PBC alone groups. No pulmonary fibrosis or central nervous system or kidney-related damage that seriously affected the prognosis of SS was found in PBC/SS patients. In addition, although SS can cause multisystem damage, patients with SS have a good clinical prognosis [18]. The above reasons might explain this finding.

The Fib-4 score is generally accepted as a noninvasive indicator of hepatic fibrosis. The Fib-4 formula incorporates age, AST levels, and platelet counts and has been tested and validated as a robust indicator of hepatic fibrosis in a variety of liver disorders, including PBC [19]. APRI scores have also been demonstrated in a number of studies to reflect the presence or absence of advanced fibrosis or cirrhosis. Thus, by incorporating both determinations and obtaining relatively consistent results, it is likely that patients with PBC alone do not have less disease progression than those with PBC/SS [20].

The prognosis of PBC patients can be accurately evaluated using the UK-PBC risk score. The best-fit model consisted of the baseline albumin and platelet count, and bilirubin, transaminase, and ALP levels, after 12 months of treatment with UDCA. In the validation cohort, the 5-, 10-, and 15-year risk scores were highly accurate [21]. Transplant-free survival could still be accurately calculated by the GLOBE score with laboratory values collected at 1 year after treatment. This model consisted of age, albumin levels, platelet counts, and bilirubin and ALP levels, after 12 months of treatment with UDCA [22]. Thus, by incorporating both models to predict outcomes and obtain relatively consistent results, concomitant SS was not associated with poorer outcomes in patients with PBC. Otherwise, the incidence of adverse events that occurred during follow-up in both groups was consistent with the above results. These findings may be explained by the good clinical prognosis of SS patients [18].

Similar to the above results, there were no differences in the rates of biochemical responses of the PBC and PBC/other EHA diseases groups. However, the PBC/other EHA diseases group had higher GLOBE scores and lower UK-PBC scores. The above results can be explained by the higher serum albumin levels in the PBC/other EHA diseases group than in the PBC alone group at baseline. PBC/other EHA diseases patients might present earlier to physicians with musculoskeletal pain than asymptomatic PBC alone patients. Additionally, the difference might be due to the higher serum albumin levels in the PBC/other EHA diseases group than in the PBC alone group at baseline. The similar ages of the two cohorts at presentation argue against this possibility as an explanation for the differences observed. In summary, the number of patients in the PBC/other EHA diseases group was too small; therefore, the results should be confirmed by others.

There are a number of limitations to this study that need to be highlighted. First, the retrospective nature of the study design contributed to missing data fields. Second, this was a single-center study, and the results should be confirmed by others. Third, the median duration of follow-up was short. Notwithstanding the above limitations, there are certain strengths of the study that warrant emphasis. Specifically, the number of patients in each cohort was appreciable for a relatively uncommon liver disorder (PBC). The biochemical response of patients after UDCA treatment for 12 months between the PBC/SS and PBC alone groups was noted for the first time in our research. Although the median duration of follow-up was short, we incorporated the UK-PBC risk score and GLOBE score to predict outcomes. Therefore, there is reason to believe that the findings in our research are credible. If possible, a larger investigation involving multiple clinical centers should be conducted in the future.

In conclusion, the results of this study suggest that concomitant SS was not associated with a poorer response or outcomes in UDCA-treated patients with PBC.

## Figures and Tables

**Table 1:** Baseline demographic and laboratory data.

	PBC(N=226)	PBC/SS(N=56)	P value
Age (years)	52.5(43.0,62.0)	51.5(45.0,58.8)	0.778
Sex, n (%)			0.123
Female	189(83.6)	52(92.9)	
Male	37(16.4)	4(7.1)	
PLT (× 10^9^/L)	127.0(84.0,192.8)	112.0(70.5,171.8)	0.244
INR	1.03(0.97,1.11)	1.01(0.97,1.08)	0.334
Alb (g/L)	41.4(35.1,44.9)	40.2(36.5,43.6)	0.369
Cr (*μ*mol/L)	60.0(52.8,71.0)	62.1(53.5,68.0)	0.635
TB (*μ*mol/L)	19.3(13.4,29.9)	16.1(12.8,25.3)	0.182
ALT (IU/L)	56.5(34.8,82.0)	62.0(40.0,88.5)	0.206
AST (IU/L)	75.0(48.0,110.0)	65.5(44.0,97.0)	0.184
ALP (IU/L)	280.5(159.8,431.3)	274.0(143.0,405.6)	0.233
GGT (IU/L)	257.5(127.8,459.0)	237.0(95.8,421.5)	0.340
Anti-AMA (%)	162(71.7)	39(69.2)	0.619
ANA (%)	210(92.9)	56(100.0)	**0.041**
IgG (g/L)	16.3(14.2,19.4)	19.3(15.5,22.2)	**0.001**
IgM (g/L)	3.6(2.3,5.9)	3.7(2.3,5.0)	0.788
Fib-4	3.6(1.9,7.1)	4.2(2.4,8.6)	0.134
APRI, splenomegaly	1.3(0.7,2.2), 71(31.4%)	1.7(0.8.2.9), 19(33.9%)	0.064, 0.718
Duration of follow-up (months)	21.0(14.0,38.3)	28.0(11.0,65.0)	0.256

Data are expressed as the median (interquartile range); PLT, platelet; INR, international normalized ratio; Alb, albumin; Cr, creatinine; TB, total bilirubin; ALT, alanine aminotransferase; AST, aspartate aminotransferase; GGT, gamma-glutamyl transferase; anti-AMA, antimitochondrial antibody; ANA, antinuclear antibody; Fib-4, fibrosis score; APRI, aspartate aminotransferase/platelet ratio index; IgG, immunoglobulin G; IgM, immunoglobulin M; SS, Sjögren's syndrome; and PBC, primary biliary cholangitis.

**Table 2:** Biochemical response to UDCA at 1 year.

	PBC(N=226)	PBC/SS(N=56)	P value
Paris-II response	144(63.7%)	34(60.7%)	0.677
Paris-I response	153(67.7%)	40(71.4%)	0.591
Toronto response	163(72.1%)	44(78.6%)	0.328
Barcelona response	147(65.0%)	41(73.0%)	0.246

SS, Sjögren's syndrome; PBC, primary biliary cholangitis.

**Table 3:** Results of liver enzyme and function testing at the end of observation.

	PBC(N=226)	PBC/SS(N=56)	P value
TB (*μ*mol/L)	16.1(11.4,27.7)	15.4(10.1,30.7)	0.392
ALT (IU/L)	29.0(19.0,45.3)	29.0(21.3,36.8)	0.557
AST (IU/L)	39.5(30.0,58.3)	36.0(30.0,54.5)	0.529
ALP (IU/L)	145.5(111.3,224.5)	131.5(103.3,189.8)	0.176
GGT (IU/L)	53.0(33.8,127.5)	59.0(30.3,94.3)	0.639
Alb (g/L)	42.8(35.0,46.5)	42.7(34.7,46.4)	0.973
PLT (×10^9^/L)	126.0(71.8,182.3)	94.0(59.8,175.3)	0.112
INR	1.0(0.9,1.2)	1.0(0.9,1.1)	0.998
Fib-4	3.0(1.8,6.8)	4.0(1.9,8.8)	0.254
APRI	0.9(0.4,1.6)	1.0(0.5,2.1)	0.489

Data are expressed as the median (interquartile range); TB, total bilirubin; ALT, alanine aminotransferase; AST, aspartate aminotransferase; GGT, gamma-glutamyl transferase; Alb, albumin; PLT, platelet; INR, international normalized ratio; Fib-4, fibrosis score; APRI, aspartate aminotransferase/platelet ratio index; SS, Sjögren's syndrome; and PBC, primary biliary cholangitis.

**Table 4:** Liver-related adverse events at the end of observation.

	PBC(N=226)	PBC/SS(N=56)	P value
Death (%)	9(4.0)	2(3.6)	0.877
HCC (%)	2(0.8)	1(1.8)	0.557
Liver transplantation (%)	2(0.8)	0(0)	0.481
Liver-related adverse events (%)	13(5.8)	3(5.4)	1.000

HCC, hepatocellular carcinoma; SS, Sjögren's syndrome; and PBC, primary biliary cholangitis.

**Table 5:** UK-PBC risk scores.

	PBC(N=226)	PBC/SS(N=56)	P value
5 years	0.054(0.027,0.175)	0.053(0.021,0.137)	0.709
10 years	0.171(0.088,0.474)	0.167(0.067,0.389)	0.710
15 years	0.295(0.158,0.698)	0.289(0.121,0.600)	0.707

Data are expressed as the median (interquartile range).

**Table 6:** GLOBE scores.

	PBC(N=226)	PBC/SS(N=56)	P value
3 years	0.896(0.689,0.958)	0.897(0.797,0.957)	0.702
5 years	0.821(0.512,0.927)	0.823(0.666,0.925)	0.702
10 years	0.588(0.165,0.814)	0.591(0.335,0.810)	0.702
15 years	0.387(0.040,0.692)	0.390(0.141,0.686)	0.702

Data are expressed as the median (interquartile range).

## Data Availability

No additional data are available.

## Supplementary Materials

Supplementary Materials Supplementary Table 1. Baseline demographic and laboratory data. Supplementary Table 2. Biochemical response to UDCA at 1 year. Supplementary Table 3. Results of liver enzyme and function testing at the end of observation. Supplementary Table 4. UK-PBC risk scores. Supplementary Table 5. GLOBE scores.
