# Supplementary material for: Concomitant Sjögren's Syndrome Was Not Associated with a Poorer Response or Outcomes in Ursodeoxycholic Acid-Treated Patients with Primary Biliary Cholangitis
Source: Can J Gastroenterol Hepatol. 2019 Jun 2;2019:7396870. doi: 10.1155/2019/7396870 (PMC6582895; doi:10.1155/2019/7396870)
Supplement: Supplementary Materials — Supplementary Table 1. Baseline demographic and laboratory data. Supplementary Table 2. Biochemical response to UDCA at 1 year. Supplementary Table 3. Results of liver enzyme and function testing at the end of observation. Supplementary Table 4. UK-PBC risk scores. Supplementary Table 5. GLOBE scores. [file 7396870.f1.docx]

**Supplementary Table 1** Baseline demographic and laboratory data

|  | PBC(N=226) | PBC/other EHA diseases(N=7) | P value |
| --- | --- | --- | --- |
| Age (years) | 52.5(43.0,62.0) | 47.0(42.0,51.0) | 0.297 |
| Sex, n (%) |  |  | 0.244 |
| female | 189(83.6) | 7(100) |  |
| male | 37(16.4) | 0(0) |  |
| PLT (× 10^9^/L) | 127.0(84.0,192.8) | 170.0(103.0,258.0) | 0.162 |
| INR | 1.03(0.97,1.11) | 1.02(0.95,1.05) | 0.482 |
| Alb (g/L) | 41.4(35.1,44.9) | 45.9(40.5,47.7) | **0.039** |
| Cr (μmol/L) | 60.0(52.8,71.0) | 51.0(50.0,70.0) | 0.507 |
| TB (μmol/L) | 19.3(13.4,29.9) | 15.4 (9.7, 24.5) | 0.162 |
| ALT (IU/L) | 56.5(34.8,82.0) | 97.0(61.0,125.0) | 0.053 |
| AST (IU/L) | 75.0(48.0,110.0) | 91.0(54.0,118.0) | 0.301 |
| ALP (IU/L) | 280.5(159.8,431.3) | 361.0(141.0,465.0) | 0.707 |
| GGT (IU/L) | 257.5(127.8,459.0) | 222.0(158.0,385.0) | 0.741 |
| anti-AMA (%) | 162(71.7) | 6(85.7) | 0.416 |
| ANA (%) | 210(92.9) | 7(100.0) | 0.467 |
| IgG (g/L) | 16.3(14.2,19.4) | 13.3(10.4,18.3) | 0.117 |
| IgM (g/L) | 3.6(2.3,5.9) | 4.0(2.6,7.2) | 0.434 |
| Fib-4 | 3.6(1.9,7.1) | 1.9(1.2,5.1) | 0.179 |
| APRI  splenomegaly | 1.3(0.7,2.2)  71(31.4%) | 1.0(0.8.2.9)  1(14.3%) | 0.699  0.335 |
| Duration of follow-up (months) | 21.0(14.0,38.3) | 35.0(12.0,71.0) | 0.211 |

Data are expressed as the median (interquartile range); PLT, platelet; INR, international normalized ratio; Alb, albumin; Cr, creatinine; TB, total bilirubin; ALT, alanine aminotransferase; AST, aspartate aminotransferase; GGT, gamma-glutamyl transferase; anti-AMA, antimitochondrial antibody; ANA, antinuclear antibody; Fib-4, fibrosis score; APRI, aspartate aminotransferase/platelet ratio index; IgG, immunoglobulin G; IgM, immunoglobulin M; EHA, extrahepatic autoimmune; PBC, primary biliary cholangitis

**Supplementary Table 2** Biochemical response to UDCA at 1 year

|  | PBC(N=226) | PBC/other EHA diseases(N=7) | P value |
| --- | --- | --- | --- |
| Paris-II response | 144(63.7%) | 4(57.1%) | 0.723 |
| Paris-I response | 153(67.7%) | 6(85.7%) | 0.314 |
| Toronto response | 163(72.1%) | 6(85.7%) | 0.429 |
| Barcelona response | 147(65.0%) | 6(85.7%) | 0.258 |

EHA, extrahepatic autoimmune; PBC, primary biliary cholangitis

**Supplementary Table 3** Results of liver enzyme and function testing at the end of observation

|  | PBC(N=226) | PBC/other EHA diseases(N=7) | P value |
| --- | --- | --- | --- |
| TB (μmol/L) | 16.1(11.4,27.7) | 12.1(9.7,16.2) | 0.143 |
| ALT (IU/L) | 29.0(19.0,45.3) | 37.0(20.0,59.0) | 0.694 |
| AST (IU/L) | 39.5(30.0,58.3) | 40.0(26.0,60.0) | 0.559 |
| ALP (IU/L) | 145.5(111.3,224.5) | 123.0(94.0,169.0) | 0.283 |
| GGT (IU/L) | 53.0(33.8,127.5) | 49.0(38.0,77.0) | 0.815 |
| Alb (g/L) | 42.8(35.0,46.5) | 46.9(45.7,48.6) | **0.010** |
| PLT (×10^9^/L) | 126.0(71.8,182.3) | 155.0(64.0,198.0) | 0.598 |
| INR | 1.0(0.9,1.2) | 1.0(0.9,1.02) | 0.105 |
| Fib-4 | 3.0(1.8,6.8) | 1.9(1.0,5.5) | 0.284 |
| APRI | 0.9(0.4,1.6) | 0.6(0.3,1.7) | 0.464 |

Data are expressed as the median (interquartile range); TB, total bilirubin; ALT, alanine aminotransferase; AST, aspartate aminotransferase; GGT, gamma-glutamyl transferase; Alb, albumin; PLT, platelet; INR, international normalized ratio; Fib-4, fibrosis score; APRI, aspartate aminotransferase/platelet ratio index; EHA, extrahepatic autoimmune; PBC, primary biliary cholangitis

**Supplementary Table 4** UK-PBC risk scores

|  | PBC(N=226) | PBC/other EHA diseases(N=7) | P value |
| --- | --- | --- | --- |
| 5 years | 0.054(0.027,0.175) | 0.022(0.014,0.057) | **0.042** |
| 10 years | 0.171(0.088,0.474) | 0.072(0.046,0.178) | **0.042** |
| 15 years | 0.295(0.158,0.698) | 0.130(0.083,0.306) | **0.042** |

Data are expressed as the median (interquartile range); EHA, extrahepatic autoimmune; PBC, primary biliary cholangitis

**Supplementary Table 5** GLOBE scores

|  | PBC(N=226) | PBC/other EHA diseases(N=7) | P value |
| --- | --- | --- | --- |
| 3 years  5 years | 0.896(0.689,0.958)  0.821(0.512,0.927) | 0.963(0.916,0.976)  0.935(0.854,0.958) | **0.034**  **0.034** |
| 10 years | 0.588(0.165,0.814) | 0.834(0.653,0.891) | **0.034** |
| 15 years | 0.387(0.040,0.692) | 0.722(0.466,0.813) | **0.034** |

Data are expressed as the median (interquartile range); EHA, extrahepatic autoimmune; PBC, primary biliary cholangitis
